# Management of Mechanical Ventilation in Decompensated Heart Failure

**DOI:** 10.3390/jcdd3040033

**Published:** 2016-12-02

**Authors:** Brooks T. Kuhn, Laura A. Bradley, Timothy M. Dempsey, Alana C. Puro, Jason Y. Adams

**Affiliations:** 1 Division of Pulmonary, Critical Care and Sleep Medicine, Department of Internal Medicine, University of California at Davis, Sacramento, CA 95818, USA; jyadams@ucdavis.edu; 2 Department of Internal Medicine, School of Medicine, University of California at Davis, Sacramento, CA 95818, USA; labradley@ucdavis.edu (L.A.B.); tdempsey@ucdavis.edu (T.M.D.); apuro@ucdavis.edu (A.C.P.)

**Keywords:** mechanical ventilation, congestive heart failure, weaning

## Abstract

Mechanical ventilation (MV) is a life-saving intervention for respiratory failure, including decompensated congestive heart failure. MV can reduce ventricular preload and afterload, decrease extra-vascular lung water, and decrease the work of breathing in heart failure. The advantages of positive pressure ventilation must be balanced with potential harm from MV: volutrauma, hyperoxia-induced injury, and difficulty assessing readiness for liberation. In this review, we will focus on cardiac, pulmonary, and broader effects of MV on patients with decompensated HF, focusing on practical considerations for management and supporting evidence.

## 1. Introduction

Heart failure (HF) affects over 5 million Americans and is the single most expensive diagnosis in the U.S. health system, accounting for 5% of the total U.S. healthcare budget [1,2]. In the U.S. annually, congestive heart failure and cardiogenic shock account for over 365,000 admissions to an intensive care unit (ICU), 80,000 of which require MV [2,3,4]. As the prevalence and cost of both heart failure (HF) and mechanical ventilation (MV) rise, adroit and nuanced management of MV is becoming increasingly important for intensivists, cardiologists, and hospitalists alike [1,5].

MV is a life-saving intervention for decompensated congestive heart failure, however, management must be guided by a knowledge of both the advantages and dangers of invasive MV in order to realize its benefits and avoid adverse effects. MV should be viewed as a tool that, if appropriately used, is a key part of the armamentarium for managing acute decompensations (Table 1).

## 2. Positive End-Expiratory Pressure

**Figure 1:**
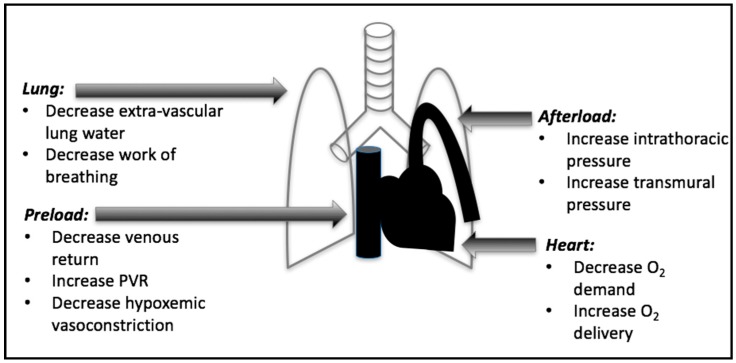
Summary of the effects of positive end expiratory pressure (PEEP).

When utilized correctly in decompensated HF, the effects of MV—specifically positive end expiratory pressure (PEEP), inspiratory support, and supplemental oxygen—can provide substantial advantages in left ventricular (LV) dysfunction and recovery. In decompensated HF, MV provides numerous benefits that include decreasing cardiac afterload leading to decreased left ventricular oxygen demand and increased cardiac output, decreasing the work of breathing which decreases cardiac output requirements, improving hypoxia-induced pulmonary vascular constriction, and improving oxygenation of the myocardium (Figure 1) [6].

### 2.1. Positive Effects of PEEP

The use of PEEP has a number of specific benefits in the setting of decompensated HF but must be used cautiously in selected circumstances. Increased intrathoracic pressure from PEEP decreases venous return and lowers preload. Mechanistically, the PEEP-mediated increase in intrathoracic pressure is transmitted to the right atrium, increasing right atrial pressure and decreasing the gradient for venous return from outside the thorax, which results in a drop in right ventricular (RV) preload and a corresponding decrease in LV preload, left atrial pressure, and pulmonary venous congestion [7]. PEEP has also been shown to decrease extra-vascular lung water directly by exerting pressure at the level of the alveoli and interstitium whereby increased intrathoracic pressure opposes pulmonary venous hydrostatic forces, resulting in movement of fluid from the alveoli and interstitium back into the vasculature [7].

PEEP can also augment cardiac output by decreasing LV afterload. LV afterload is proportional to LV end-diastolic volume and LV systolic transmural pressure [7]. LV systolic transmural pressure is defined as the gradient between the intrathoracic (largely determined by pleural pressure) and intracardiac (LV systolic) pressures. Use of PEEP increases pleural pressure, thereby reducing transmural pressure, with a resultant reduction in LV afterload. In healthy patients, the effects of PEEP on reducing afterload are felt to have minimal effects on CO. In contrast, CO in patients with LV systolic dysfunction is extremely sensitive to changes in afterload making the application of PEEP an important component of afterload optimization [8]. Clinical studies support this theory in practice; one study demonstrated that in patients with elevated pulmonary capillary wedge pressure (PCWP) (especially those with PCWP of >18) the initiation of PEEP led to an increase in CO [9]. Grace and colleagues showed improvement in RAP, PCWP, and cardiac index with the application of PEEP in patients in the surgical ICU with post-CABG LV dysfunction requiring mechanical ventilation [10]. While multiple studies have shown improvements in surrogate hemodynamic markers with the use of PEEP in patients with heart failure with reduced ejection fraction (HFrEF), data supporting the use of PEEP to improve hard clinical outcomes are somewhat limited. The clinical benefits of PEEP were suggested in a case series of 28 patients with cardiogenic shock requiring intra-aortic balloon pump support that found that the subset of patients that were intubated and mechanically ventilated with 10 cm H_2_O of PEEP were more likely to survive to discharge, had improved hemodynamic indices, and required lower doses of dobutamine than those receiving oxygen supplementation alone [9]. The potential beneficial effects of PEEP in HF can also be inferred from randomized controlled trials examining the use of non-invasive continuous positive airway pressure (CPAP). In a large, recent meta-analysis that examined 15 randomized, controlled trials (RCT) comparing CPAP to standard medical management alone in patients with acute decompensated HF, Vital et al. found a 53% reduction in the need for endotracheal intubation, shorter ICU length of stay (−1 day), and a 40% decrease in in-hospital mortality with the use of continuous positive airway pressure ventilation [10].

### 2.2. Negative Effects of PEEP

Despite evidence of the potential benefits of PEEP in patients with decompensated HF, the use of PEEP needs to be weighed against potential harms. Increasing PEEP in patients with low end-diastolic volume can decrease venous return, resulting in inadequate preload, reduced CO, and end-organ hypoperfusion [7]. High levels of PEEP may also increase pulmonary vascular resistance, which may be especially important in cases of biventricular failure or right heart-predominant cardiogenic shock [8]. Patients with decompensated HF presenting with signs of hypo- or euvolemia, such as those with acute myocardial infarction, may be particularly susceptible to PEEP-mediated reductions in preload and so PEEP should be increased cautiously in these populations. In addition, rapid increases in PEEP in the setting of aggressive diuresis may contribute to transient under-filling of either ventricle which can also precipitate hypotension.

### 2.3. PEEP Summary

In the early years of clinical practice with mechanical ventilation, PEEP was believed to have a negative effect on cardiac output and tissue perfusion based on data extrapolated from animal models without cardiac disease [6]. Over the past two decades, however, numerous studies in animal models of HF and in patients with decompensated HF have shown that PEEP can in fact can lead to improved LV function, cardiac output, and oxygen delivery including in those with cardiogenic shock [11]. Current evidence is convincing that if applied judiciously, the combined effects of PEEP can be beneficial in the management of patients with decompensated HF [7,12,13]. To maximize potential benefits of PEEP, we recommend starting with a PEEP of 5 cm H_2_O with incremental increases of 2–3 cm of H_2_O every 15–30 min as needed, guided by careful monitoring of hemodynamics and indices of end-organ perfusion in order to identify the optimal, non-injurious PEEP level.

## 3. Inspiratory Support and Tidal Volume

The inspiratory support offered by MV is an often-overlooked advantage in patients with decompensated heart failure and cardiogenic chock in particular. When patients are in respiratory distress, respiratory muscles can require as much as 16% of the cardiac output [6,14]. Furthermore, patients with heart failure may achieve less than 50% of the maximal cardiac output attained by healthy individuals under stress [15]. The inspiratory support provided by MV can unload the work of breathing required to ventilate a less compliant, edematous lung, allowing limited cardiac output to better meet the metabolic needs of non-pulmonary organ systems. In this regard, even small amounts of pressure support have been shown to have profound effects with as little as 5 cm H_2_O of pressure support resulting in a decrease of over 30% in the work of breathing [16].

### 3.1. Pulmonary Edema

Cardiogenic pulmonary edema develops when the hydrostatic pressure gradient increases between the pulmonary capillaries and surrounding interstitial space, leading to fluid accumulating in the extravascular space. With elevated interstitial hydrostatic pressure, fluid enters the alveoli [17,18]. Increased capillary hydrostatic pressure occurs in pulmonary venous hypertension (e.g., as with LV failure) and with elevated pulmonary blood flow (e.g., as with fluid overload) [17,18]. The resulting pulmonary edema leads to decreased lung compliance, hypoxemia, and increased work of breathing [19]. In this regard, Perlman et al. demonstrated that fluid-filled alveoli decreased in size, placing mechanical stress on the neighboring air-filled alveoli that substantially reduced overall lung compliance [18]. The mechanical changes in pulmonary compliance resulting from interstitial and alveolar edema highlight the importance of providing adequate amounts of inspiratory support to offset the metabolic demands of the associated increased work of breathing. We suggest the use of assist controlled modes of mechanical ventilation (e.g., assist control-volume control or assist control-pressure control) in the early phases of mechanical ventilation to provide a guaranteed amount of inspiratory support.

### 3.2. Tidal Volume

While there have been limited studies specifically addressing the selection of tidal volumes for patients with decompensated HF receiving MV, numerous studies have shown the clinical benefits of low tidal volume ventilation between 6–8 mL per kilogram of predicted body weight (PBW) in various disease states including the acute respiratory distress syndrome (ARDS), intraabdominal surgery, high risk cardiac surgeries, and trauma [20,21,22,23]. While the lifesaving advantages of MV for patients with acute respiratory failure are clear, potentially injurious effects of MV (e.g., barotrauma, volutrauma, atelectrauma) may place patients with even relatively “healthy” lungs at increased risk for adverse outcomes from ventilator-induced lung injury (VILI) [24]. While previous authors have suggested that a tidal volume of ≤10 mL per kilogram of PBW is acceptable for patients without risk factors for lung injury, we feel that the weight of clinical evidence supports the use of a target tidal volume of 6–8 mL per kilogram of PBW for all patients receiving MV unless contraindicated by special circumstances (e.g., severe acidemia, increased intracranial pressure) [23,25,26].

Transpulmonary pressure (TPP) is defined as the alveolar pressure minus the intrapleural pressure, and represents the alveolar distending pressure that is “felt” by the lung [27]. Since intrapleural pressure is not currently practical to measure in most ICUs, plateau pressure—measured during a brief end-inspiratory hold—is used as a surrogate for TPP and measures the total static recoil forces of the respiratory system exerted by the lungs, pleural space, chest wall, and abdomen. It is important to recognize that a number of extrapulmonary conditions common in patients with decompensated HF can contribute to the plateau pressure and may render the metric a poor indicator of alveolar distention, including pleural effusions, ascites, and anasarca. It is also important to recognize that patient effort can lead to inaccurate measurement of plateau and bedside clinicians should ensure that patient efforts during inspiratory holds do not result in erroneous measurements. In general, maintaining plateau pressure below 25–30 cm H_2_O limits the risk of barotrauma, which can result in pneumothorax and pneumomediastinum, and limits excessive alveolar distention, which can lead to ventilator-induced lung injury [23,24].

## 4. Hypoxemic Vasoconstriction and Supplemental Oxygen

Alveolar hypoxia and acidemia reduce nitric oxide production, leading to pulmonary vasoconstriction [28]. In healthy individuals, this allows better-ventilated areas of the lung to be perfused, thereby limiting pulmonary shunting and improving ventilation/perfusion matching [8,15]. In decompensated heart failure, edema leads to alveolar hypoxia and spontaneous alveolar collapse, which may trigger excessive hypoxic pulmonary vasoconstriction, and consequently increased PVR and decreased RV stroke volume. MV recruits and oxygenates collapsed alveoli via PEEP, thereby mitigating these adverse hemodynamic effects [8,28].

Increasing the amount of supplemental oxygen, controlled by the fraction of inhaled O_2_ (FiO_2_), is the fastest intervention to improve acute hypoxemia. Similar to the management of PEEP and tidal volume, excessive supplemental oxygen can lead to adverse hemodynamic consequences including ventilation-perfusion mismatch, impaired cardiac relaxation, and increased LV filling pressures in patients with HF independent of their ventilation and sympathetic activity [29,30]. FiO_2_ in excess of 50% can lead to oxygen free radical formation, which has been shown to worsen cardiac apoptosis, lead to intracellular calcium overload, and cause cardiac hypertrophy [31,32]. In animal models, FiO_2_ above 50% has been shown to cause coronary vasoconstriction, decreased stroke volume and cardiac output, and foci of myocardial necrosis [33]. A study of post-cardiac arrest patients found hyperoxia (defined as partial pressure of oxygen greater than 300 mm Hg within one hour of arrest) was associated with higher in-hospital mortality, postulated to be due to an increase in sensitivity to oxygen toxicity during reperfusion [34]. Careful titration of supplemental oxygen should be used target an oxygen saturation of 92%–96% and FiO_2_ should ideally be ≤50% to avoid free radical damage (Table 1).

## 5. Neurohumoral Modulation

Neurohormonal modulation—the balance of sympathetic and parasympathetic activity regulating vascular tone, heart rate, and contractility—in decompensated HF is regulated by receptors in both the heart and the lung. Sympathetic stimulation predominates in HFrEF, leading to profound systemic vasoconstriction mediated by the renin-angiotensin system and endogenous vasopressin and norepinephrine [35]. Positive pressure mechanical ventilation can affect neurohumoral modulation through a variety of mechanisms: parasympathetic activity from lung stretch receptors; increased intrathoracic pressure affecting cardiac baroreceptors; and release of endogenous norepinephrine [36].

### 5.1. Hering–Breueur Reflex

The Hering–Breuer reflex (i.e., pulmonary stretch receptors in smooth muscle signal the respiratory centers in the pons and medulla to inhibit further inhalation), recently proven to be in humans, limits excessive tidal volumes [37]. These stretch receptors stimulate parasympathetic activity with larger breaths. Recent experience with neurally-adjusted ventilatory assist (NAVA), a mechanical ventilation mode that uses a diaphragmatic electromyogram to detect inspiratory effort and provide ventilator assistance synchronized with patient effort, has proven the Hering–Breuer reflex is active in humans [38]. While this may theoretically suggest that larger tidal volumes could be beneficial in HFrEF, no clinical data exist to support such a practice, and it is likely that the recognized benefits of low tidal volume ventilation with regard to prevention of ventilator-induced lung injury outweigh any potentially favorable effects of large tidal volumes on neurohormonal regulation. Furthermore, extreme tidal volumes of >15 mL/kg of PBW have been shown to dramatically decrease heart rate and arterial vascular tone, leading to decreased cardiac output and shock via sympathetic withdrawal [39].

### 5.2. Autonomic Tone

Chronic heart failure is characterized by a net increase in sympathetic nerve activity from numerous contributing factors (both advantageous and deleterious), the predominant contributor being cardiopulmonary baroreflex mechanisms [40,41]. In patients with chronic HF, sympathetic outflow from lung and ventricular stretch receptors and decreased lung compliance from pulmonary edema lead more rapid, smaller breaths [42]. The stimulation of vagal afferent receptors in the lung with larger tidal volumes and a higher functional residual capacity is postulated to be one of the mechanisms for reduced sympathetic activity in positive pressure ventilation [43]. The addition of continuous positive airway pressure, analogous to the use of PEEP, has been shown by Butler et al. to increase heart rate variability through augmentation of parasympathetic activity [44]. Frazier et al. implicated a role for decreasing sympathetic tone with fully supported MV by showing an increase in serum catecholamines during the decrease in ventilator support associated with spontaneous breathing trials [45]. While more research is clearly needed, these data highlight the potential impact of MV on neurohormonal regulation in HF.

## 6. Cheyne-Stokes Respiration

Cheyne-Stokes respiration (CSR) is common in heart failure with reduced ejection fraction (HFrEF) with an incidence of 25%–40%, correlating with disease severity [46]. CSR is a centrally-mediated abnormal breathing pattern marked by alternating hyper- and hypo-ventilation with a crescendo-diminuendo pattern. The cardiovascular effects of CSR are complex. CSR may be beneficial when large tidal volumes stimulate parasympathetic activity [47]. However, CSR has also been associated with increased sympathetic tone, apnea-related hypoxemia leading to diastolic dysfunction and arrhythmias, impaired REM sleep, and increased mortality [48,49].

Data regarding the management of mechanical ventilation in CSR are all derived from studies of non-invasive positive pressure ventilation (NIPPV). Despite a number of randomized, controlled trials, however, the role of non-invasive mechanical ventilation in the management of CSR remains unclear. The CANPAP randomized, controlled trial of CPAP in patients with HFrEF and central sleep apnea demonstrated improved left ventricular ejection fraction (2.2% ± 5.4%), improved nocturnal oxygenation, and lower endogenous norepinephrine levels, but no change in survival [50]. Adaptive servo ventilation (ASV) is a form of noninvasive positive pressure ventilation designed specifically for CSR, augmenting ventilation during hypopnea and reducing ventilatory support during tachypnea. Kasai et al. demonstrated improved treatment compliance with ASV compared to CPAP, as well as improved sleep quality and improvement in left ventricular ejection fraction (32.0% ± 7.9% to 37.8% ± 9.1%; *p* < 0.001) [51]. The SAVIOR-C study compared ASV to standard medical therapy (not CPAP) in patient with HFrEF and an LVEF < 40%. There were no significant differences in BNP and left ventricular ejection fraction, but quality of life and functional status improved significantly [52]. More recently, the SERVE-HF randomized controlled trial of ASV in patients with CSA and HFrEF with an LVEF < 45% found increases in all-cause (hazard ration 1.28) and cardiovascular mortality (hazard ration 1.34), with no improvement in quality of life or functional status [53]. The SERVE-HF study had higher minute ventilation (>10 L per minute) and higher inspiratory pressures than SAVIOUR-C, potentially explaining the higher rates of arrhythmia and cardiovascular death [53]. Taken together, these data argue against the routine use of ASV in patients with chronic HFrEF.

### Cheyne-Stokes Respirations in Mechanically Ventilated Patients

For patients with CSR requiring invasive MV, there is a paucity of data to guide care. Extrapolating the logic behind ASV to invasive MV management, it is our recommendation to use modes of MV that provide a consistent level of inspiratory support (assist control-volume control, assist control-pressure control, or pressure support ventilation). Dynamic pressure-targeted modes such as proportional assist ventilation, volume support, and pressure-regulated volume control, should generally be avoided in patients with CSR since constantly changing respiratory drive may result in inappropriate ventilator settings and patient-ventilator asynchrony. Spontaneous breathing trials may be stopped prematurely due to apnea; therefore, it is our recommendation that apnea alarm thresholds be elongated, assuming no associated hemodynamic instability. While the optimal management of mechanical ventilation in CSR remains unclear, it is important to remember that treatment of heart failure with standard medical therapies will often improve CSR.

## 7. Liberation and Weaning from Mechanical Ventilation

While the rapid shallow breathing index and spontaneous breathing trials (SBT) are the most ubiquitous indices to assess readiness for extubation, not all SBTs are performed with the same ventilator settings. In clinical practice, there is a spectrum of PEEP and pressure support levels used while performing SBTs; from T-piece trials (performed without PEEP or supplemental ventilator support) to “minimal ventilator settings” (often with a PEEP of 5 cm H_2_O and pressure support ≤8 CM H_2_O). While the latter can appear to be a trivial amount of support, in patients with decompensated HF even small amounts of pressure support and PEEP can have significant hemodynamic effects. Removal of seemingly minimal amounts of PEEP can result in rapid deterioration of LV function leading to pulmonary edema [54]. Similarly, even small amounts of pressure support can have large effects on reducing the work of breathing. For example, the addition of 5 cm H_2_O of pressure support decreases inspiratory work by 31% to 38%; 10 cm H_2_O of pressure support decreases work by 46% to 60% [16]. In this regard, Cabello et al. compared three different levels of SBT support in patients that had failed a previous T-piece trial and in whom pulmonary artery catheters were felt necessary for clinical management: pressure support ventilation (PSV) with PEEP, PSV without PEEP, and T-piece. Most patients passed the PSV and PEEP tests, whereas all failed the T-piece trial. Swan-Ganz catheter measurements in the T-piece group indicated higher pulmonary artery occlusion pressures during the trial [55]. In 2014 a Cochrane review examined the success of minimal support SBT versus T-piece trials in unselected patients undergoing MV [56]. Though the authors deemed that the quality of evidence was low due to “limitations in the design of the studies and imprecision in the effect estimates,” the overall conclusion was that there was no difference in success of ventilation weaning, need for reintubation, or ICU mortality. While not supported as standard practice for all patients requiring MV, patients with impaired cardiac function require a higher threshold for extubation. For this reason, we frequently perform spontaneous breathing trials in patients with marginal cardiac function without any supplemental support (i.e., T-piece trial) to assess the patient’s ability to tolerate fully unsupported breathing. T-piece trials in this patient population may reveal acute pulmonary edema, arrhythmia, or hemodynamic instability that were not evident during minimal support trials due to the favorable effects of PEEP and/or pressure support on preload, afterload, and work of breathing. T-piece trials in patients with impaired cardiac function may unmask the need for further optimization of preload and afterload, both before and after extubation, in order to prevent re-intubation.

Despite passing a spontaneous breathing test, approximately 10%–20% of patients fail and need reintubation [57]. Reintubation is independently associated with mortality and mortality rates range from 25%–50% in patients requiring re-intubation [57,58]. Patients older than 65 years and those with chronic respiratory or cardiac disease are at high risk for failure [59].

### 7.1. Predictors of Extubation Failure

Heart failure and fluid balance are predictors of failure and should be optimized prior to planned extubation. Cabello and colleagues identified heart failure as the cause of 42% of failures of spontaneous breathing trials in a large cohort of medical ICU patients [55]. Another study found positive fluid balance the day before extubation, regardless of pre-existing HF, to predict extubation failure [58]. Brain natriuretic peptide (BNP) can be used as a surrogate for detecting SBT failure due to heart failure. A study by Zapata et al. found a BNP greater than 263 nanograms per liter or an absolute change in BNP by greater than 48 nanograms per liter to predict SBT failure [60]. A randomized clinical trial showed a BNP-driven fluid management protocol decreased the duration of mechanical ventilation, especially in patients with chronic HFrEF [61]. Papanikoloau and colleagues used Doppler echocardiography and found that *E/E_m_* ratios greater than 7.8 predicted successful extubation [62]. Ultrasound is also used in the lung to identify the presence of B-lines—a sign of interstitial edema—which is a validated, sensitive measure of extra-vascular lung water. Lung ultrasound has a low accuracy, but high negative predictive value (86%) for predicting extubation failure [63]. Another, simpler modality to evaluate for optimization of fluid status prior to liberation is the passive leg raise (PLR). The PLR establishes preload dependence and is positive if cardiac index (CI) increases by >10% during passively raising the lower limbs by 45 degrees [64]. A recent trial showed a negative PLR prior to SBT predicted failure due to cardiac dysfunction with a sensitivity of 97% and specificity of 81% [64].

### 7.2. Optimizing Peri-Extubation Care

To prevent extubation failure in HF, non-invasive positive pressure ventilation (NIPPV) can be applied prophylactically. Nava et al. found amongst patients at risk of re-intubation, including those with heart failure, that the prophylactic use of NIPPV for at least 8 h per day in the first 48 h following extubation lead to a 10% relative risk reduction in ICU mortality, likely mediated by a reduction in the need of reintubation [65]. Ferrer et al. decreased extubation failure and ICU mortality with early NIPPV in patients at high risk of extubation failure [66]. It is important to note that this benefit has not been proven in an unselected population [57]. In contrast to the prophylactic use of NIPPV in patients at high risk of extubation failure, use of NIPPV to rescue patients with post-extubation respiratory failure should generally be avoided in favor of prompt re-intubation. In this regard, a clinical trial by Esteban et al. randomized unselected patients with acute respiratory failure in the 48 h after extubation to either standard therapy (supplemental oxygen, respiratory physiotherapy, bronchodilators) or to NIPPV [67]. There was no difference in the rate of reintubation, but there was increased mortality in the noninvasive ventilation group.

It is the authors’ opinion that patients requiring inotropic support in whom liberation from mechanical ventilation is being considered merit particularly close attention due to restricted ability to increase cardiac output under physiologic stress. For this reason, we frequently perform spontaneous breathing trials in patients with marginal cardiac function without any supplemental support (e.g., T-piece trial) to assess the ability to tolerate fully unsupported breathing. Careful optimization of preload, afterload, and inotropic support (in selected patients) both before and after extubation is paramount to preventing re-intubation.

## 8. Conclusions

The hemodynamic benefits of MV are often-overlooked in patients with decompensated congestive heart failure and cardiogenic shock in particular. Favorable effects of MV in acute decompensated HF include reducing ventricular preload and afterload, decreasing extra-vascular lung water, and decreasing the work of breathing and its associated cardiac output requirements. The appropriate use of MV should be seen as an important adjunctive therapy in the initial stabilization and management of patients with decompensated HF and acute respiratory failure. Patients with acute decompensated HF require special attention to ventilator weaning and liberation from MV since HF is well recognized as a risk factor for extubation failure and the need for re-intubation. Spontaneous breathing trials without ventilator support (i.e., T-piece trials) may reveal the need for further medical optimization of preload, afterload, and contractility prior to planned extubation since removal of even a seemingly trivial amount of support can result in hemodynamic instability, flash pulmonary edema, and rapid decompensation. Prophylactic use of non-invasive positive pressure ventilation immediately upon extubation may prevent re-intubation in patients with marginal cardiac function but non-invasive ventilation should not be thought of as a rescue therapy for patients who develop acute respiratory failure after extubation since this practice has been associated with increased mortality compared to medical therapy alone with prompt re-intubation if needed. When carefully applied, both invasive mechanical ventilation and non-invasive positive pressure ventilation should be considered important tools in the successful management of respiratory failure in patients with acute decompensated HF.

## Figures and Tables

**Table 1:** Recommended ventilator settings for patients with heart failure with reduced ejection fraction.

Setting	Recommended Initial Ventilator Settings
PEEP	Titrate to adequate oxygenation, work of breathing, and hemodynamics. Recommend preferential use of PEEP for oxygenation if hemodynamically beneficial.
Tidal Volume	8 cc/kg predicted body weight
FiO_2_	Titrate to SpO_2_ 90%–94%. Recommend rapid de-escalation of FiO_2_ after intubation.
Plateau pressure	Maintain below 30 cm H_2_O. Consider alternative diagnoses if plateau rises above 30.
Respiratory rate	In conjunction with tidal volume, titrate to maintain normal pH (7.35–7.45) and pCO_2_ (35–45 mm Hg)
Inattention to changing needs	Provide the minimal ventilator support to support physiologic stability. MV requires frequent re-evaluation and titration.
